# Thermally Reversible Polymeric Networks from Vegetable Oils

**DOI:** 10.3390/polym12081708

**Published:** 2020-07-30

**Authors:** Frita Yuliati, Jennifer Hong, Keshia S. Indriadi, Francesco Picchioni, Ranjita K. Bose

**Affiliations:** 1 Laboratory for Polymer Technology, Agency for the Assessment and Application of Technology, Jalan M.H. Thamrin no. 8, Jakarta 10340, Indonesia; frita.yuliati@bppt.go.id; 2 Department of Chemical Engineering, University of Groningen, Nijenborgh 4, 9747 AG Groningen, The Netherlands; jennifer.hong905@gmail.com (J.H.); keshia.saradima@gmail.com (K.S.I.); f.picchioni@rug.nl (F.P.)

**Keywords:** jatropha oil, sunflower oil, Diels–Alder, thermally reversible networks

## Abstract

Low cross-link density thermally reversible networks were successfully synthesized from jatropha and sunflower oils. The oils were epoxidized and subsequently reacted with furfurylamine to attach furan groups onto the triglycerides, preferably at the epoxide sites rather than at the ester ones. Under the same reaction conditions, the modified jatropha oil retained the triglyceride structure more efficiently than its sunflower-based counterpart, i.e., the ester aminolysis reaction was less relevant for the jatropha oil. These furan-modified oils were then reacted with mixtures of aliphatic and aromatic bismaleimides, viz. 1,12-bismaleimido dodecane and 1,1′-(methylenedi-4,1-phenylene)bismaleimide, resulting in a series of polymers with T_g_ ranging between 3.6 and 19.8 °C. Changes in the chemical structure and mechanical properties during recurrent thermal cycles suggested that the Diels–Alder and retro-Diels–Alder reactions occurred. However, the reversibility was reduced over the thermal cycles due to several possible causes. There are indications that the maleimide groups were homopolymerized and the Diels–Alder adducts were aromatized, leading to irreversibly cross-linked polymers. Two of the polymers were successfully applied as adhesives without modifications. This result demonstrates one of the potential applications of these polymers.

## 1. Introduction

Increasing concerns about the unstable prices of petroleum resources along with the questions regarding its availability in the future have initiated a growing interest in the use of renewable resources [1,2,3]. Various vegetable oils are produced on a large scale worldwide. Most of these oils are consumed as food and feed, and to a lesser extent used as raw materials for fuels and chemicals, including a wide application in the polymer industry [4,5].

Vegetable oils are extensively utilized in different polymeric materials such as the classic use as oil paint medium (drying oil) [6,7,8,9] to the production of contemporary materials as polyesters [10,11] and polyurethanes [12,13]. Numerous researches have employed different chemical reactions to synthesize various polymers from the oils, among others, cationic polymerization [14], metathesis [15,16], thiol-ene [17], and thiol-yne [18], Michael addition [19], ATRP [20], and Diels–Alder (DA) reactions [2,21,22,23,24]. The DA reaction is a very interesting option for polymerization because it can be reversed at an elevated temperature. Applying this reaction to cross-linked polymers offers the possibility of reprocessing the material, which is not an option with the conventional ones [25]. These reprocessable materials possess distinctive capabilities of self-healing and recyclability, making them advantageous in practical, economical, and environmental aspects.

The DA reaction occurs between a conjugated diene with a dienophile to form a six-membered hydroaromatic or heteroaromatic ring, with the formation of two σ bonds at the expense of two π bonds of the precursors [26]. Application of this reaction on vegetable oils and their derivatives is not new and was intended for various applications. Some vegetable oil structures include dienes which can be modified into conjugated ones. Safflower oil was isomerized to arrange the C–C double bonds into conjugated configuration and used in DA reactions, in earlier works dating back to 1971 and 1972 [27,28,29]. Esters of the modified oil were successfully reacted with maleic anhydride, styrene, and acrylic acid as dienophiles [27,28,29]. Another interesting raw material is tung oil, which naturally contains conjugated trienes. This oil was reacted with maleic anhydride at 150 °C for 2 h, producing a high yield of DA product [30]. Another research group reported the polymerization of the same oil with 1,1′-(methylenedi-4,1-phenylene)bismaleimide with varied compositions, producing polymers with T_g_ varying from 111–150 °C and tensile modulus in the range of 1.67 to 2.58 GPa [31]. However, another work found that similar polymers synthesized from tung oil and different bismaleimides did not demonstrate the occurrence of retro-Diels–Alder (rDA) reaction, and thus could not be reprocessed under heating [23]. This might at least suggest the presence of side reactions besides rDA at relatively high temperatures.

Other synthetic routes were developed in utilizing different vegetable oils for DA reaction-based polymers. A popular way of doing so is by appending groups containing dienes and dienophiles to vegetable oil derivatives and subsequently combining them in a DA reaction into linear or cross-linked polymers. An example to this route is the modification of 10-undecenoic acid and undecenoic alcohol into monomers bearing two furans or a furan and a protected maleimide. The first monomer was reacted with 1,6-bismaleimidohexane while the second was reacted by itself after removing the protecting group. The reaction in solution took place over several days to obtain high conversions with the reversed reaction consumed similar amount of time [32], and this expected to proceed faster when using higher concentrations [21]. Multifuranic structures can also be built from undecenoic alcohol by combining three molecules of the alcohol into a triglyceride and attaching furans to the terminal alkenes. The trifuranic monomer was cross-linked with bismaleimide in a reaction monitored by NMR. It was found that high yield was obtained and the reversibility feature occurred in the system [2].

Most vegetable oils do not possess conjugated dienes or terminal alkenes. Attaching furans into the oil structure can be performed by first modifying the unsaturations to make it more reactive, for example, by epoxidation. Epoxidized oils can further be modified to yield acrylated vegetable oils such as the commercially available acrylated epoxidized soybean oil (AESO). In a study, furans were successfully attached to AESO via Michael addition, leading to triglycerides comprising three furan moieties. The furan-functionalized molecule was reacted with bismaleimide to obtain a cross-linked polymer. This polymer underwent rDA reaction at temperatures ranging between 103 and 134 °C [33].

Other studies attempted to use a shorter route, attaching furans directly to epoxidized oils by reacting them with furfurylamine. The amine group also reacted with esters, resulting in linear structures comprising one or two furan moieties instead of triglycerides with at least three furans. After reactions with bismaleimides, brittle polymers were obtained as the products [24,34]. In our previous work [35], the conditions for a similar functionalization of jatropha oil were optimized, attaining high epoxide conversion while retaining a majority of esters. When the product was cross-linked with bismaleimides, tougher polymers can be expected as a result of longer hydrocarbon chains functioning as the “backbone” of the polymers with relatively lower cross-link density.

**Figure 1:**
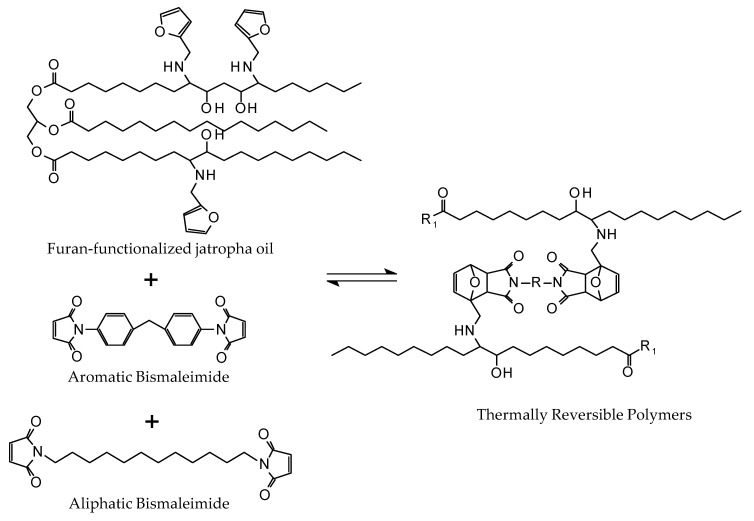
Synthesis of a thermally reversible polymer by reacting furan-functionalized jatropha oil with a mixture of bismaleimides.

In this work, we cross-linked the furan-functionalized jatropha oil with combinations of two different bismaleimides in order to obtain polymers with different rigidities (Figure 1). The first is a commercially available bismaleimide comprising two aromatic rings and the second has an aliphatic chain instead of the rings. The same procedure was applied to sunflower oil to understand to what extent a different level of functionalization influences the properties of the resultant polymers. The occurrence of DA and r-DA reactions was also investigated by observing changes in chemical structure and mechanical properties throughout thermal treatments.

## 2. Materials and Methods

### 2.1. Materials

Jatropha curcas oil (JO) was purchased from Dilligent BV, Eersel, Netherlands. Sunflower oil was purchased from Jumbo supermarket. Hydrogen peroxide solution (30%), formic acid (≥98%), sodium chloride (≥99%), furfurylamine (≥99%), 1,12-diaminododecane (98%), tris(2-aminoethyl)amine (96%), maleic anhydride (99%), triethylamine (≥99.5%), acetic anhydride (≥99%), DMF anhydrous (99.8%), silica gel 60 A 230-400#, silica gel technical grade 40 6-12#, 1,1′-(methylenedi-4,1-phenylene)bismaleimide (95%), and 2,5-dimethylfuran 99% were obtained from Sigma Aldrich (Munich, Germany). Peroxide test strips (0.5, 2, 5, 10, 25 mg/L H_2_O_2_) were procured from Merck (Darmstadt, Germany). Toluene (99.5%) and dichloromethane (99.5%) were purchased from Macron Fine Chemicals (Deventer, The Netherlands), whereas sodium chloride (≥99%) and lithium bromide (≥98%) were obtained from Fluka (Landsmeer, The Netherlands). A representative all-purpose mounting glue was purchased from Bison (Rotterdam, Netherlands), while aluminum sheets (20 × 20 × 0.01 cm^3^) were purchased from aluminiumfolie.nl (Gorinchem, The Netherlands).

### 2.2. Methods

#### 2.2.1. Epoxidation of Jatropha and Sunflower Oils

Epoxidation of the vegetable oils was performed according to a previous work [24] with some modification. Here, 20 mL (0.02 mol) of the oil was dissolved in 100 mL of toluene and heated to 70 °C. Into the solution, formic acid and 30% H_2_O_2_ solution (12 mL, 0.53 mol, and 150 mL, 1.47 mol, respectively) were added dropwise in one hour while being stirred, and the stirring was continued for 24 h. The mixture was separated by using a separating funnel. The organic phase was collected and excess hydrogen peroxide was removed by liquid-liquid extraction by using 150 mL of 5% NaCl solution. The concentration of the remaining hydrogen peroxide was tested by using peroxide test strips. Toluene was evaporated by using a rotary evaporator, and the remaining water was absorbed by using 6–12 # silica gel.

#### 2.2.2. Furan-functionalization of the Epoxidized Oils

Furan-derivatization of epoxidized oils was performed based on a reported method [35]. 5 g (0.005 mol) of purified epoxidized oil and furfurylamine were reacted with the molar ratio between the epoxides and furfurylamine of 1:1. Lithium bromide was introduced at the amount of 100 mol % of the triglyceride. The mixture was stirred at 400 rpm, at the temperature of 115 °C for 24 h. The products were dissolved in chloroform, then the unreacted furfurylamine was washed with 250 mL of MiliQ water. The water and organic phase were separated by using a separating funnel, and the organic phase was used in the polymerization afterwards.

#### 2.2.3. Synthesis of 1,12-Bismaleimido Dodecane

The 1,12-bismaleimido dodecane was synthesized according to a reported procedure [36,37]. 7.3 g (0.075 mol) of maleic anhydride was dissolved in 20 mL of dimethylformamide and heated to 80 °C. 5.0 g (0.025 mol) of 1,12-diaminododecane was added into the solution and stirred for 1 h until a clear solution was formed. Acetic anhydride (15.3 g, 0.15 mol), triethylamine (1.0 g, 0.0099 mol), and nickel(II)acetate (0.10 g, 0.00057 mol) were added into the solution. The stirring was continued for 30 min. Then, 20 mL of mili-Q water was added into the solution, and the solvent was removed by using a rotary evaporator. The residue was dissolved in 200 mL of dichloromethane and shaken with 30 g of silica gel for 5 min. Silica gel was removed by filtration, after which chloroform was removed by vacuum evaporation. The residue was dissolved in 50 mL of boiling ethanol, before the solution was kept at 0 °C overnight. Crystals were formed and retrieved by filtration.

#### 2.2.4. Model Reactions

Model reactions between furan and maleimide groups were performed by using dimethylfuran and each of the aromatic and aliphatic bismaleimide. 0.8 g (0.008 mol) of dimethylfuran was mixed with 0.1 g (0.0003 mol) of the aromatic bismaleimide or 0.1 g (0.0003 mol) of the aliphatic bismaleimide. The mixtures were stirred by using a magnetic stirrer at 400 rpm, 50 °C, for 24 h. The occurrence of DA and rDA reactions was observed by FTIR identification during two heating-cooling cycles between 50–110 °C.

#### 2.2.5. Cross-Linking via the Diels-Alder Reaction

A mixture of aromatic and aliphatic bismaleimide (with compositions according to Table 1) was added to a 10% solution of furan-functionalized vegetable oil in chloroform, with the molar ratio of furans and maleimides of 1:1. This mixture was stirred at 400 rpm at 50 °C for 24 h. Chloroform was then removed from the solution via evaporation. The products were kept in a vacuum oven at 50 °C for 24 h to remove the remaining solvent and further cure the polymers. The curing process was continued at room temperature for two weeks.

#### 2.2.6. Characterization

The compositions of vegetable oils, epoxidized oils, and furan-functionalized oils were analyzed by using ^1^H-NMR in a Varian Oxford 300 MHz NMR (Agilent, Santa Clara, CA, USA) with deuterated chloroform as a solvent. The occurrence of the DA and rDA reactions in the products of the model reaction as well as the polymers were identified by using an attenuated total reflectance (ATR) crystal, in an IRT Racer-100 Shimadzu FTIR (Kyoto, Japan), equipped with Specac Golden Gate ATR Top and West 6100+ temperature controller (Philadelphia, PA, USA). Spectra were recorded in the absorbance mode between 500 and 4000 cm^−1^, with 64 repetitions and a resolution of 8 cm^−1^. The measurement was performed every 20 °C during two heating-cooling cycles. For the model compound, the preferred temperature range was between 50 °C and 110 °C, while for the polymer it was between 50 °C and 150 °C. The signals assigned to the DA adducts and maleimides were deconvoluted and the areas were calculated by using Origin 8.1 software (Northampton, MA, USA). The spectra of the model compounds were normalized to the C=O signal of the maleimides at 1700 cm^−1^, while those of the polymers to the unreacted alkyl signal at 2845 cm^−1^.

The thermo-mechanical properties of the polymers were measured by using a Perkin Elmer DMA 8000 (Waltham, MA, USA), using the single cantilever mode. The measurements were performed in a temperature sweep experiments between 5 and 55 °C. Furthermore, the DA and rDA reactions were observed by measuring the polymers in three heating and cooling cycles between 0 and 150 °C. For these experiments, the polymer samples were held in between thin pockets made of stainless steel. Due to the use of these material pockets the absolute values of the modulus obtained include the contribution from the polymer samples as well as the outer stainless-steel pocket. The samples were loaded at room temperature, then the temperature was increased to 150 °C and cooled to 0 °C. All DMA measurements were performed with 0.005 mm amplitude, at 1 Hz frequency. The samples used in the DMA measurement were collected in 4 mL vials and subjected to a gel content measurement according to a reported method [38] with some modification. THF was added to these vials at 20 times the mass of the samples. The vials were closed with the lids and let sit at room temperature for 24 h, after which the mixtures were filtered. The retained solid was dried and weighed by using an analytical balance. The amount of insoluble polymer was calculated by using Equation (1).

(1) $$\%\;Insoluble=\frac{m_{0}-m_{1}}{m_{0}}\times 100\%$$

#### 2.2.7. Polymer Performance as Adhesive Test

**Figure 2:**
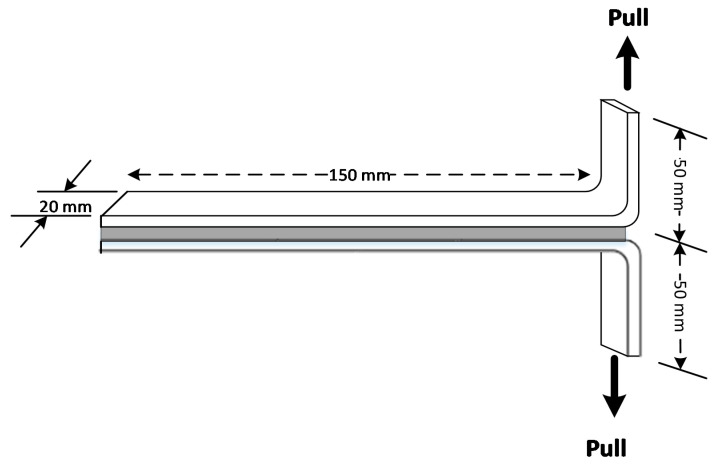
T-peel testing scheme according to ASTM D 1876-08 with some modifications.

The polymers J-40-60 and SF-80-20 were applied to adhere two aluminum sheets. The adhesive performance was measured according to ASTM method D1876-08 [39] with some modifications. 3 g of the polymer was shaped into sheets by compression molding at 70 °C for 5 min, at a pressure of 25 kN. A polymer sheet (15 × 20 cm^2^, instead of 15.2 × 24.1 cm^2^ according to the ASTM method) was placed between the two aluminum sheets (20 × 20 cm^2^, instead of 15.2 × 30.5 cm^2^ stated in the testing standard). The sandwich was compressed at 70 °C for 5 min at a pressure of 25 kN. The adhesive was allowed to cure at room temperature for 2 days. The sheets were cut into stripes (2 × 20 cm^2^, as opposed to 2.5 × 30.5 cm^2^ stated in the testing standard). Nine specimens were tested for each adhesive formulation. The peel resistance of the adhesive was measured by a universal test device shown in Figure 2. The speed of the machine was set at 254 mm/min. The data collected was load versus position. The peel resistance was calculated as the average load (kg) divided by the stripe width (cm). Due to slight deviations of the testing protocol with respect to the ASTM standard (vide supra), a commercial all-purpose mounting glue was tested as a comparison in order to correctly benchmark our results. The adhesive was applied to the aluminum sheets by using a scraper. A uniform load was applied to the adhered sheets for 2 days. The sandwich was prepared for a peel test using the same method as used for the samples prepared in this study.

## 3. Results and Discussion

### 3.1. Epoxidation of the Vegetable Oils

**Figure 3:**
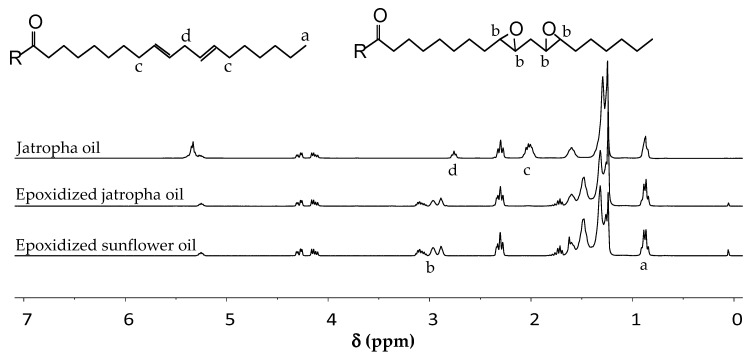
Proton NMR spectra of epoxidized jatropha and sunflower oils compared to jatropha oil.

The first step in this work is epoxidation of the vegetable oils according to a previous work [40], which entails the use of an excessive H_2_O_2_ to ensure a complete conversion of the C–C double bonds. The vegetable oils were analyzed by using ^1^H NMR, before and after epoxidation (Figure 3). Jatropha and sunflower oils have similar structures with a different number of unsaturations. Therefore, their NMR spectra are similar, only differing in the intensity of signals related to vinylic and allylic protons. Full unsaturation conversion was recognized by the disappearance of signals related to the double bonds. The signal of the vinylic proton at 5.34 ppm is slightly overlapping with a signal representing a proton on the glyceride group. Therefore, other signals related to allylic protons (2.01 ppm) and bisallylic ones (2.75 ppm) were used in determining the progress of the reaction. The average number of epoxide rings in a triglyceride molecule was determined by the area below the epoxide signals (2.82–3.18 ppm), with terminal methyl signals (0.88 ppm) used as an internal standard. It was found that the epoxidized jatropha and sunflower oils contained on average 3.27 and 4.36 epoxides in each triglyceride molecules. As displayed in Figure 3, the structure of epoxidized jatropha and sunflower oils are similar. However, the epoxidized sunflower oil spectrum contains stronger epoxide signal than jatropha, due to a higher number of epoxides in each triglyceride.

### 3.2. Furan-Functionalization of the Epoxidized Oil

**Figure 4:**
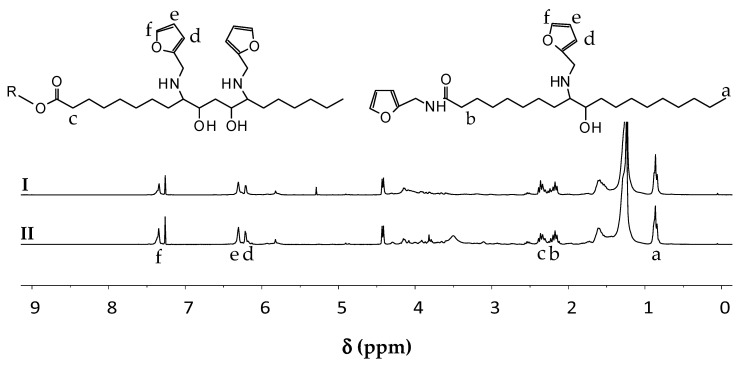
Proton NMR spectra of: (I) furan-functionalized jatropha oil and (II) furan-functionalized sunflower oil.

The epoxidized oils were reacted with furfurylamine in order to attach furans to the epoxide sites via a ring opening reaction. The amines also reacted with the esters, thus an unwanted ester aminolysis reaction also occurred. In a previous work [35], the reaction was optimized with epoxidized jatropha oil as a reactant. The optimum condition was found to be at 115 °C for 24 h, with a 1:1 molar ratio of epoxides to furfurylamine and 100% mol of LiBr loading, according to which the unwanted side reactions were minimized. Full epoxide conversion was difficult to achieve probably because of the presence, in some of the fatty acid chains, of two neighboring epoxide groups, with one of them being therefore sterically hindered [21]. According to NMR spectra of the products (Figure 4), furan-functionalized jatropha oil used in this work had on average 2.36 and 0.73 units of esters and epoxides remaining, with an average of 2.57 furans attached to each triglyceride molecule. When the same reaction condition was applied to the epoxidized sunflower oil, it was found that the epoxide conversion was lower and the ester conversion was higher. The sunflower-based product had on average 2.00 and 1.27 units of esters and epoxides remained, with 3.42 furans attached to each triglyceride. These results suggest that the products contain a mixture of mono-, di-, and triglycerides and fatty chains containing furans in their structures. The jatropha-based product contained a larger portion of triglycerides than the sunflower-based product. This distribution in chain length and functionalization of the precursor stems from and dovetails the original structure of the vegetable oil. Nevertheless, it must be noted here that the functionalization degree is in all cases high enough (>2) to ensure the formation of a polymeric network after curing.

### 3.3. Model Reactions

**Figure 5:**
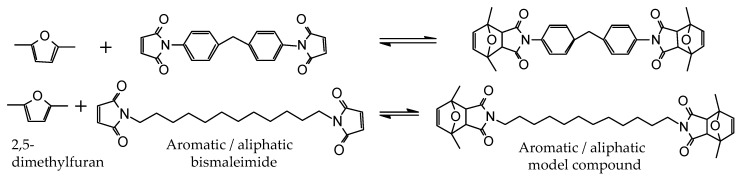
Model reaction scheme.

In order to synthesize the thermally reversible polymers, furan-functionalized oils were reacted with bismaleimides via the DA reaction. In this work, aromatic and aliphatic bismaleimides were mixed in order to tailor the mechanical properties of the polymers. Indeed, a previous work carried out on ethylene/propylene copolymers functionalized with furan and cross-linked in the same way (i.e., with bismaleimide) demonstrated a detectable influence of the cross-linker structure on the final properties [37]. To provide more insight to the DA reaction between furan groups and each of the bismaleimides, 2,5-dimethylfuran as a model furan compound was reacted with each of the aromatic and aliphatic bismaleimides (Figure 5).

**Figure 6:**
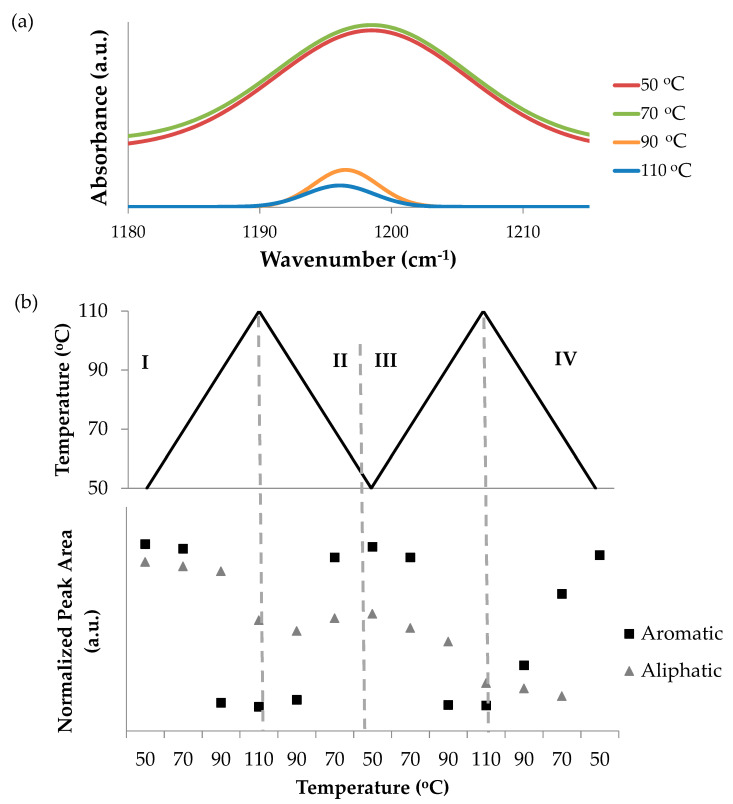
(**a**) Changes in the FTIR peak assigned to the C–O–C group of the DA adduct in the first heating cycle of 2,5-dimethylfuran with the aromatic bismaleimide during the first heating; and (**b**) changes in the peak area of the DA adduct peak of reaction products with aromatic and aliphatic bismaleimide.

Each of the bismaleimides was mixed with excessive 2,5-dimetylfuran without an additional solvent, and stirred at 50 °C for 24 h. The extent of the DA and rDA reactions and their dependency towards temperature were investigated by using FTIR spectrometry during two heating-cooling cycles between 50–110 °C (Supplementary Figure S1). This range of temperature was preferred because it was expected to accommodate both reactions while avoiding the degradation of the model compounds which was found to occur above 110 °C (not shown for brevity), and minimizing the evaporation of 2,5-dimethylfuran (boiling point of 92–94 °C). The peak assigned to the C–O–C group of DA adduct [38,41] was recognized at around 1197 cm^−1^ for the reaction with aromatic bismaleimide, and around 1170 cm^−1^ for the one with aliphatic bismaleimide (Figure 6). These peaks were divided by the area of the peak related to the asymmetric C=O stretching vibration of maleimide and DA adduct [42,43] at 1700 cm^−1^. In the case of the aliphatic model, this calculation led to very small values. In order to make the observation easier, these numbers were further normalized to the initial peak areas of each sample.

Bismaleimides present in the form of powders and they are soluble in 2,5-dimethylfuran. After the mixtures were stirred at 50 °C for 24 h, the aromatic model compound appeared as an opaque pasty material, while the aliphatic model compound was a transparent brownish liquid. The DA reaction occurred during stirring, as the DA adduct signal was visible at the beginning of the identification by using FTIR. The changing peak areas attributed to the DA signal of the aromatic and aliphatic models are displayed in Figure 6. Both the samples demonstrate changes that indicate the occurrence of the DA and rDA reactions. During heating (zone I and III), the DA adduct signal weakened, while it was strengthened during cooling (zone II and IV).

The changes of the DA adduct signal of the aromatic model was more dramatic, implying that both DA and rDA reactions occurred with high yield. Although the mixture was heated to above the boiling point of 2,5-dimethylfuran, it did not evaporate much, probably because it was trapped in the structure of the pasty material.

The aliphatic model also demonstrated reversibility, as the peak area of the DA adduct decreased during heating (zone I and III), and increased during cooling (zone II). However, the overall trend of this signal change was negative, implying that the amount of adduct decreased over the thermal cycles. The physical appearance of the aliphatic model compound did not change during the stirring process and the intensity of the DA adduct signal was low, which might indicate that DA reaction proceeded to a low extent. Free 2,5-dimethylfuran might evaporate during the thermal cycles, leaving a decreasing amount available for the DA reaction.

### 3.4. Synthesis and Characterization of Thermally Reversible Networks

**Figure 7:**
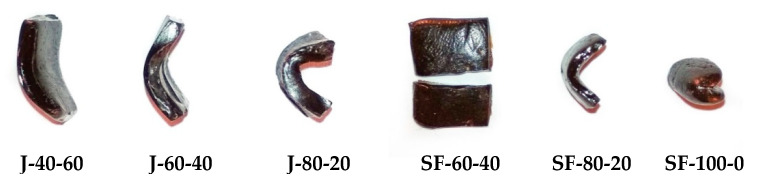
Polymers synthesized with different bismaleimide composition. The codes J and SF refer jatropha or sunflower oil, the numbers describe the percentage of aliphatic and aromatic bismaleimides respectively.

Thermally reversible networks were synthesized by reacting the furan-functionalized oils with a mixture of the bismaleimides at different compositions, as explained in Table 1. The formation of the polymeric materials is expected to result from DA reaction between the furan groups in the modified oil and the bismaleimides. Polymers with different rigidities were retrieved as the product of these reactions (Figure 7). As expected, higher concentration of aliphatic bismaleimide used in the reaction generated more flexible polymers [37]. With the same composition of bismaleimides, jatropha-based polymers were more flexible than their sunflower ones. Sunflower-based polymer with 60% of aliphatic bismaleimide was too rigid to be bent, while the similar jatropha-based polymer can form a curve from bending. This difference arises from the higher content of triglyceride structure retained in the modified jatropha oil. Higher triglyceride contents induced longer backbone and lower cross-linking density, and hence higher flexibility of the polymers. The storage modulus of the polymers at 20 °C range between 1.56 × 10^7^ Pa to 8.80 × 10^7^ Pa, except for SF-60-40 that was too brittle to undergo thermo-mechanical measurement. The storage modulus curves of the polymers can be found in Supplementary Figure S2.

**Figure 8:**
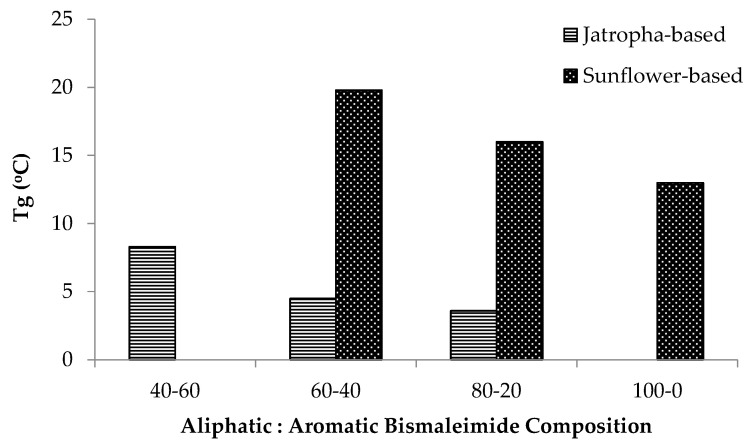
Glass transition temperature of jatropha- and sunflower-based polymers according to the bismaleimide compositions.

The difference of the polymer structure also influenced their T_g_, as described in Figure 8. Sunflower-based polymers possess a higher T_g_ than the jatropha-based ones due to the difference in their cross-link densities. Higher cross-link density means that the molecular segments between cross-links are shorter, thus the segmental mobility is hindered. Higher aromatic bismaleimide content also results in higher T_g_ of the polymers because its structure is more rigid and inflexible compared to the aliphatic bismaleimide. Both the higher cross-link density and the aromatic content increase the amount of thermal energy needed to enable molecular mobility under dynamic conditions, thus increasing the temperature at which the molecules become more mobile [44].

The composition of the aliphatic and aromatic bismaleimides was found to influence the mechanical properties of the polymers. In a previous work, furan-derivatized jatropha oil was cross-linked with only the aromatic bismaleimide, generating materials too brittle for practical applications [40]. Aliphatic bismaleimide was found to increase the flexibility of the materials, which is in agreement with a recent work where the bismaleimide was used to cross-link EPM rubber [37]. It was also found that the composition of the bismaleimides can be adjusted to obtain polymers with the desired flexibility.

**Figure 9:**
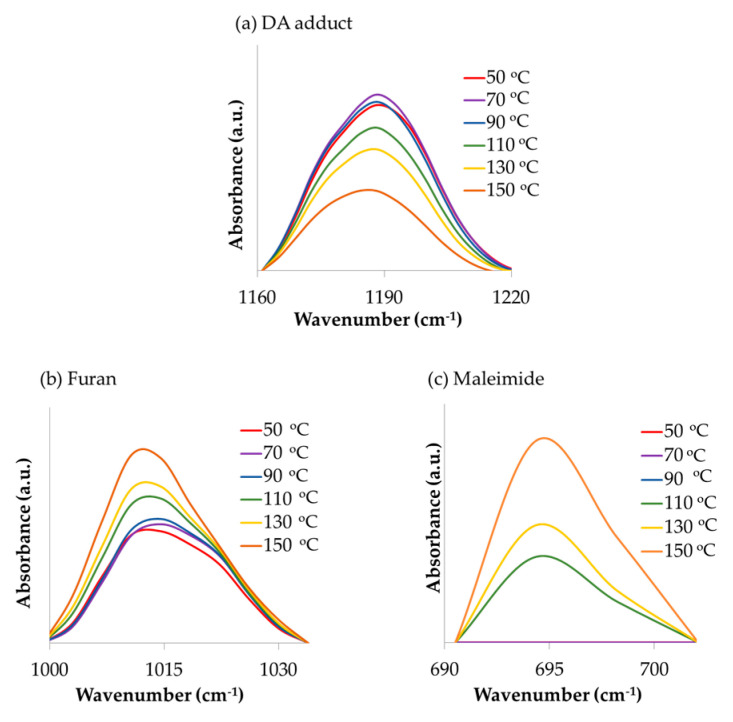
FTIR peaks of polymer J-40-60 taken at different temperatures during heating from 50 °C to 150 °C, assigned to the (**a**): DA adducts, (**b**): furans, and (**c**): maleimides.

**Figure 10:**
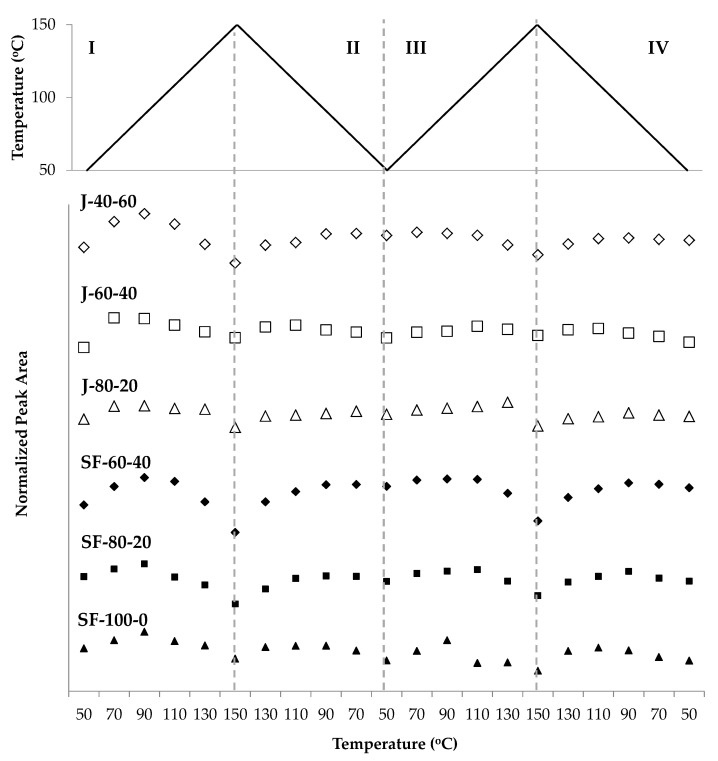
The changing peak areas attributed to the C–O–C group in the DA adduct at 1180 cm^−1^ during heating and cooling of the jatropha oil- and sunflower oil—based polymers, normalized to the peak area of the unreacted methyl at 2854 cm^−1^_._

The thermo-reversibility of the polymers was confirmed by using FTIR during 2 heating-cooling cycles. The spectra were normalized to the unreacted alkyl signal [45,46] at 2854 cm^−1^. Figure 9 displays a series of FTIR spectra taken during the first heating of the J-40-60 polymer (zone I in Figure 10). The signal attributed to the ether group in the DA adduct [38,47] at around 1185 cm^−1^ decreased with increasing temperature, while the furan ring breathing signal [47] at 1011 cm^−1^ and maleimide ring deformation signal [41] at 693 cm^−1^ increased. The observation of the DA and rDA reactions occurring in the samples was focused on the changes of these signals.

Further changes on the DA adduct during two cycles of heating and cooling of the polymers can be observed in Figure 10. It is worthwhile to note that the intensities of this peak decreased as the portion of aliphatic bismaleimide in the polymers was increased. Since the areas and the changes of peaks related to the furans and maleimides are similar for all samples, it can be expected that the differences in DA adduct peaks arise from the structural difference between the aliphatic and aromatic bismaleimides. In order to render the observations in the peak at 1185 cm^−1^ quantitatively, the curves in Figure 10 were normalized so that all polymers exhibit similar intensities.

**Figure 11:**
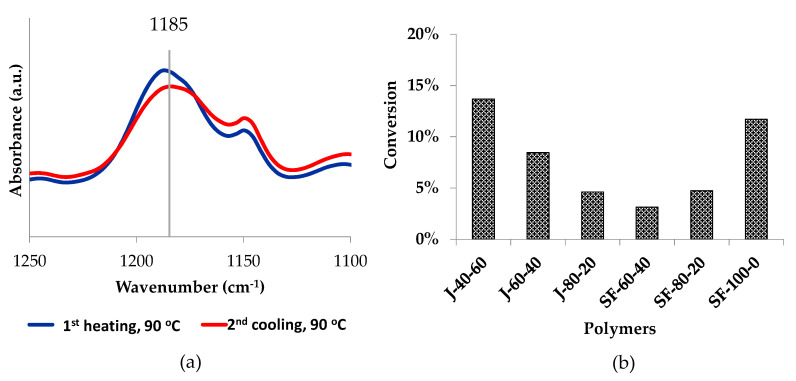
FTIR curves of polymer J-40-60 describing changes in signals related to the DA adduct, measured at 90 °C on the first heating and second cooling cycles (**a**). The peak at 1185 cm^−1^ corresponds to the C-O-C group, while the one at 1070 cm^−1^ represents the furan in-plane C–H deformation. The bar graph (**b**) displays how much of the peak areas were reduced during the measurement.

The area of the DA adduct peak at 1185 cm^−1^ decreased during heating (zone I and III) and increased during cooling (zone II and IV). At the beginning of zone I, the peak area increased, indicating that the DA reaction took place until the temperature reached 70 °C or 90 °C. After reaching the maximum value, the areas gradually decreased while the temperature was increased to 150 °C, implying that the rDA reaction occurred. The areas increased again during cooling (zone II) and reached their new maximum at 70 °C on the second heating, then slightly decreased at 50 °C, then bounced back up at 70 °C. The trend was repeated in the second heating-cooling cycle, implying that the reactions can be performed repeatedly. However, the maximum peak area decreased with more cycles, implying that the amount of DA adduct was reduced. The peak area corresponds to the ether group of the DA adduct, thus the decrease of this signal might suggest that a portion of the ether was no longer present, probably because the adduct was aromatized [38,48]. As shown in Figure 11, the decrease of this signal is visible when comparing its maximum intensity at 90 °C, in the first heating and second cooling cycles. However, there are other possible explanations to this trend, as revealed by other data obtained in this study.

An interesting phenomenon is clearly observed in Figure 10. At 50 °C, the DA adduct was expected to be at its maximum amount as a consequence of the DA reaction, however, the correlating peak area at this temperature is always lower than at 70 °C. It appears that the rDA reaction was dominant at 50 °C. A possible explanation for this phenomenon is the existence of two stereoisomers of the DA adduct, the endo and exo isomers. The endo isomer undergoes rDA reaction at lower temperature [49,50,51], in our case, at around 50 °C, resulting in the decrease of DA adduct peak area. During heating to 70 °C, the free furan and maleimide groups subsequently reacted to form exo isomers [49], increasing the DA adduct peak. Further heating triggered the rDA reaction of the exo adducts starting at above 90 °C in most of the polymer samples.

**Figure 12:**
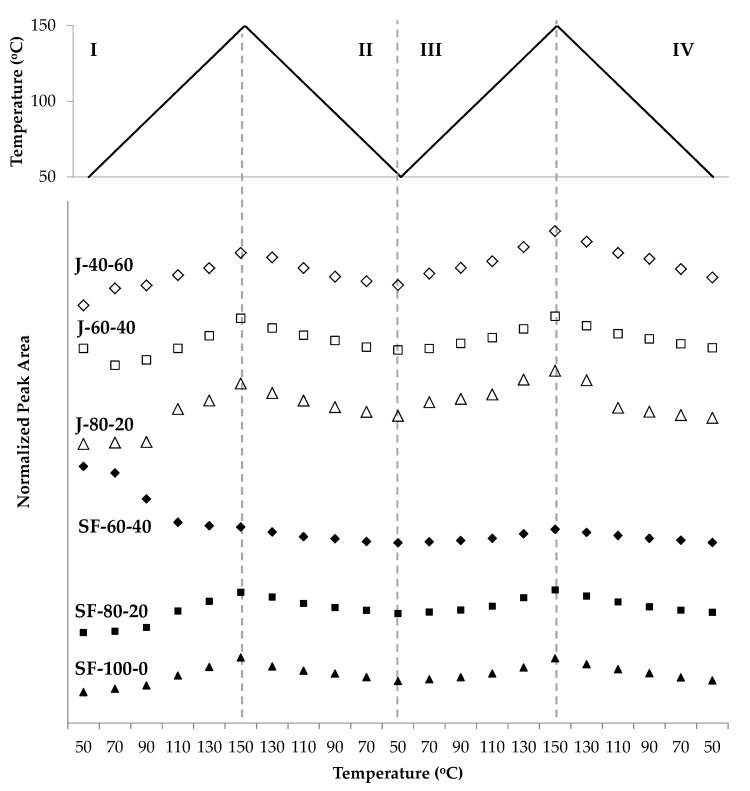
The changing area of a peak related to furans at 1012 cm^−1^ during two heating-cooling cycles of the jatropha oil- and the sunflower oil-based polymers.

The changes of the furan peak area at around 1011 cm^−1^ support the idea of DA and rDA reactions occurring during the measurement (Figure 12). The furan peak areas increased during heating (zone I and III), implying that the furans were formed in the rDA reaction. The peak area reached its maximum at 150 °C and was reduced during cooling, with the same trend took place in the second cycle. Measured furan peak area in the second cycle (zone III) was slightly higher than in the first (zone I), indicating that the amount of unreacted furan was higher than that in the first. This condition is in agreement with the trend in Figure 10, indicating that less DA adduct was formed in the second heating-cooling cycle.

**Figure 13:**
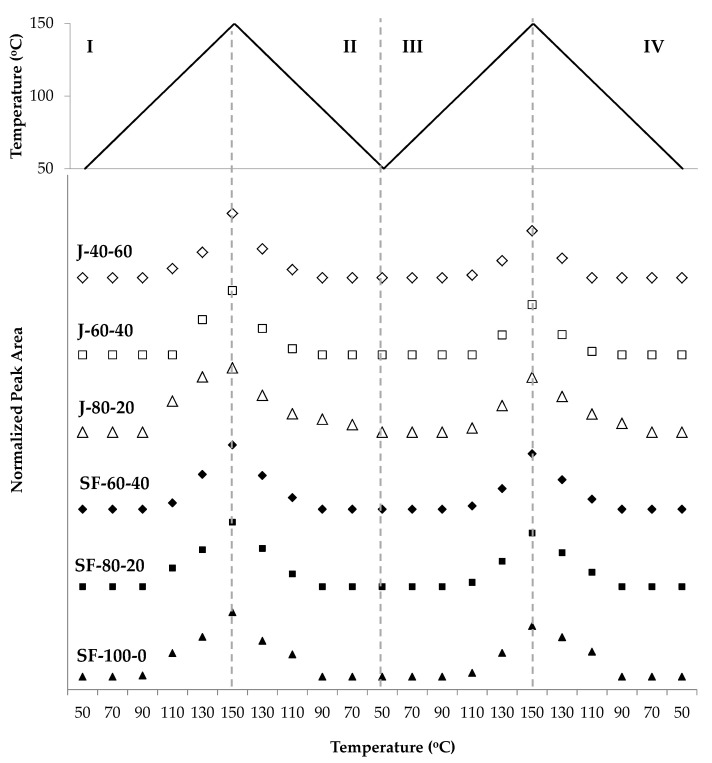
The increase and decrease of peak areas assigned to maleimides during heating and cooling of the jatropha oil- and the sunflower oil-based polymers.

The maleimide signal located at around 693 cm^−1^ of all polymers also demonstrated the thermo-reversibility of the polymers (Figure 13). The signal increased during heating (zone I and III), revealing that the maleimides were formed in the rDA reaction. The signal decreased during cooling (zone II and IV), implying that the maleimides were consumed in the DA reaction. This signal was visible at temperatures above 90 °C for most polymers, indicating that the rDA reaction was dominant in these temperatures. In contrast to the furan signal, the maleimide signal was not visible below 90 °C, which can be interpreted as a complete or nearly-complete conversion of the maleimides. On the other hand, the furan signal was always visible, revealing that they were not entirely consumed in the DA reaction.

**Figure 14:**
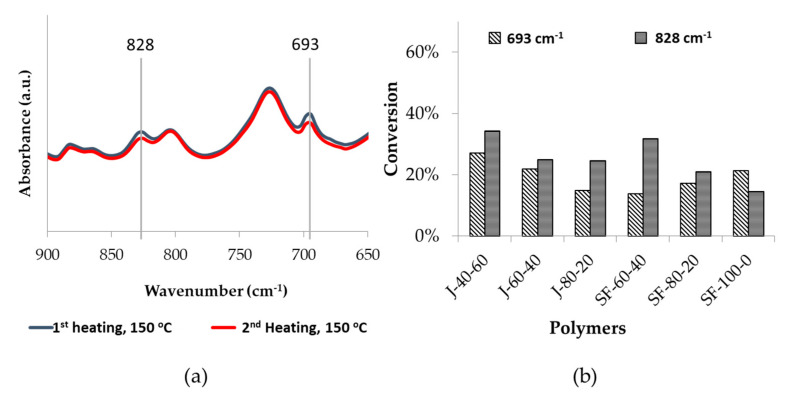
FTIR curves of polymer J-80-20 displaying changes in signals related to the maleimides, measured at 150 °C on the first heating and second cooling cycles (**a**). The peak at 828 cm^−1^ corresponds to the C–C double bond, while the one at 693 cm^−1^ represents the ring deformation. The bar graph explains how much of the peak areas were reduced during the measurement (**b**).

Another observed trend in Figure 13 is that the maleimide peak area was lower in the second cycle than the first. This fact supports the hypothesis that the DA reaction was less prominent in the second cycle. The amount of bismaleimide was reduced, thus there was less of this group available for the DA reaction, hence the number of unreacted furans increased. Decreasing maleimides amount might be caused by homopolymerization of this group [52]. Maleimides can react with one another above their melting point [53], and the experiment temperature reached close or above their melting points (156–158 °C for the aromatic bismaleimide [54] and 103 °C for the aliphatic bismaleimide [37]). Another evidence of maleimide homopolymerization is the decreasing peak area of signals at 828 cm^−1^, attributed to the C=C bond of the maleimide ring [55,56], measured at 150 °C in the first and second heating of the measurement. A sample of the decreasing peak areas of maleimides-related signals is given in Figure 14.

Polymer reversibility was also observed during measurements by using DMA. Since the polymers were deformed at temperatures above 55 °C, the measurements were performed by using material pockets. This device was designed to enable the measurement of thermo-mechanical transitions of powders in their native forms by using DMA [57], therefore it is also applicable in non-self-supporting samples such as polymers above their softening point. The absolute value of storage, loss modulus, and tan δ did not reveal the real properties of the material due to the use of material pockets, however, the transition temperature data is reliable. Therefore, the tan δ curves are used to observe the transition temperatures of the polymers during the measurement. The values of tan δ are denoted in arbitrary units (a.u.).

**Figure 15:**
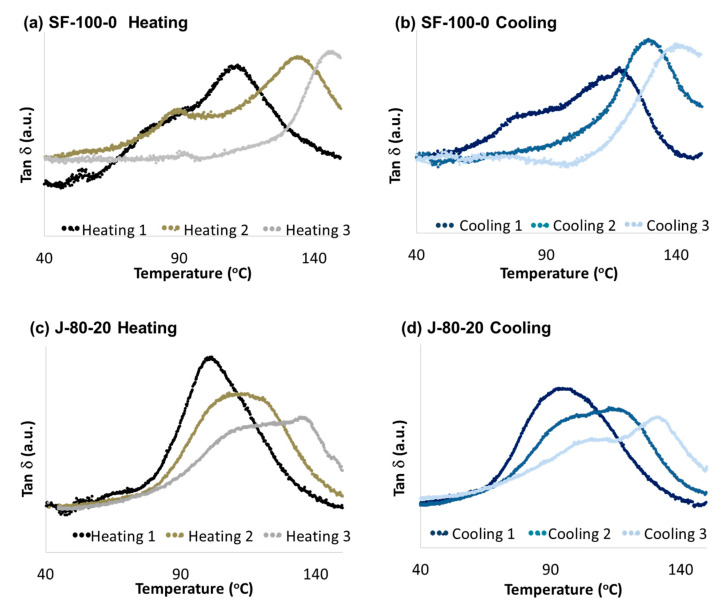
DMA curves showing changing temperatures of the rDA and DA reactions obtained in measurements with 3 heating-cooling cycles. The curves were taken during (**a**): SF-100-0 heating cycles, (**b**): SF-100-0 cooling cycles, (**c**): J-80-20 heating cycles, and (**d**): J-80-20 cooling cycles.

**Figure 16:**
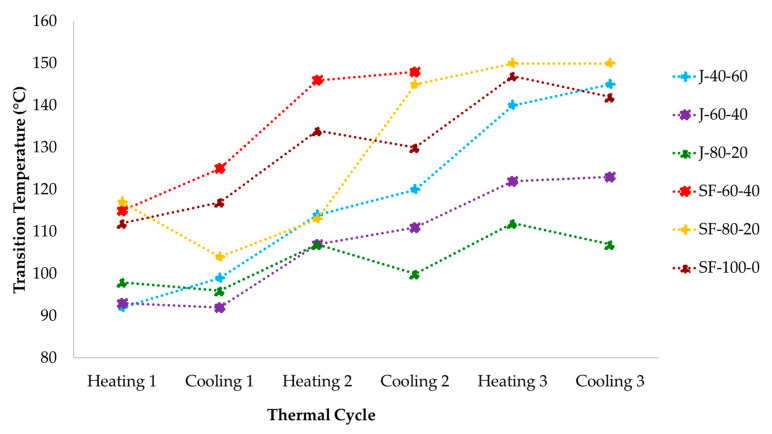
Transition temperatures observed in DMA measurements with 3 heating-cooling cycles. Lines are guides for the eye.

Figure 15 demonstrates the transitions observed during the DMA measurement of polymer J-80-20 and SF-100-0 with three heating-cooling cycles. The peaks of tan δ demonstrate the rDA reaction during heating (left) and DA reaction during cooling (right). The peaks of the curves shift to the right, demonstrating the increasing tempeartures at which DA and rDA reaction took place with the thermal cycles. This tendency might be related to the aromatization of the DA adduct or homopolymeryzation of the maleimides [52], both producing irreversible cross-links [48,58,59,60]. These networks reduced the mobility of the materials, and thus restraining the DA and rDA reactions to occur. The trend also applied to other polymer samples, which are summarized in Figure 16.

Some shoulders appeared at temperatures between 60 °C and 90 °C on both of tan δ curves of SF-100-0 and heating of J-80-20 in Figure 13. These shoulders indicate that there are other transitions than the aforementioned DA and rDA reactions. Since these transition occurred at lower temperatures than the main transitions, it can be expected that these are related to the DA and rDA reactions of the endo adduct [49,50]. During heating, the shoulders revealed that there was a slight drop of the storage modulus, which can be related to the rDA reaction of the endo DA adduct into furans and maleimides. During cooling, the shoulders might suggest the formation of endo DA adducts. These events may correlate with the trend in Figure 10, in which the amount of the adduct was reduced at around 50 °C. However, the FTIR spectra demonstrate the re-forming of DA adducts during heating, while the DMA curves display a continuous decrease in storage modulus (Figure S3 of the Supplementary Materials). There are several possible explanations for this event, i.e., the DA reaction took place at a lower extent under dynamic condition, or the influence of the DA reaction on the storage modulus was very low compared to the softening of the material due to a temperature increase.

Figure 16 includes the summary of the transition temperatures from the DMA measurement, which are related to the DA and rDA reactions. All polymers demonstrated similar trend of ascending transition temperatures with increasing number of heating-cooling cycles. This trend might indicate that by exposing the polymers to more thermal cycles, an increasing portion of the polymers became irreversibly cross-linked, thus inhibit both reactions. This tendency is in agreement with the data extracted from FTIR spectra on Figure 6 describing that the amount of the adducts were reduced during the measurement. In general, the samples prepared from sunflower oil showed slightly higher transition temperatures than those derived from jatropha oil. The higher cross-link density of the sunflower-based polymer might require a higher temperature for the DA and rDA reactions to occur [61].

**Figure 17:**
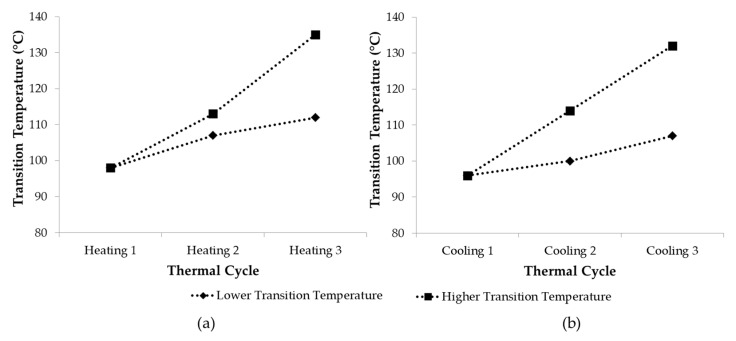
Two transition temperatures observed during the DMA measurement of polymer J-80-20 (**a**) related to the rDA reaction during the 2nd and 3rd heating; and (**b**) related to the DA reaction during 2nd and 3rd cooling. Lines are guides for the eye.

Polymers with aliphatic bismaleimide content below 80% showed only one transition temperature on each cycles. When the aliphatic bismaleimide content was increased to 80%, two transitions were observed (Figure 15). However, the polymer cross-linked with 100% aliphatic bismaleimide returned to only having one transition on each cycle. This finding might suggest that at a high concentration of the aliphatic bismaleimide, each of the bismaleimide underwent the DA chemistry separately. The separation was observed at the second and third cycles during the measurement of polymer J-80-20 (Figure 17). On the contrary, the separation of polymer SF-80-20 was found from the early cycles of the measurement (not shown for brevity). In the third heating and cooling cycle of SF-80-20, the transitions at higher temperatures were not observed because they seemed to occur above the measurement range.

**Figure 18:**
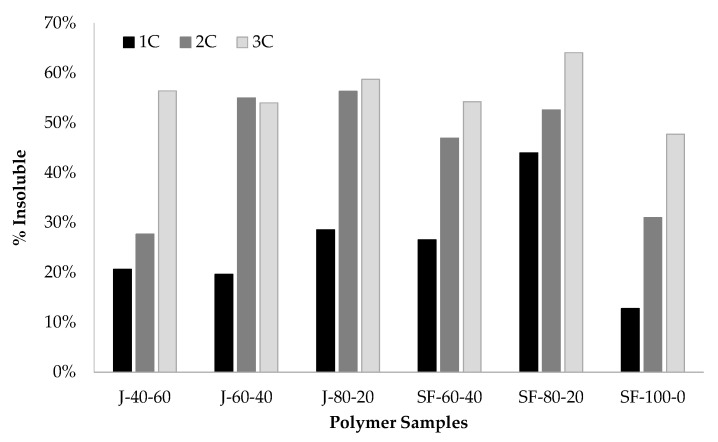
The percentage of insoluble polymer samples after DMA measurements with 1, 2, and 3 heating-cooling cycles.

The samples used in DMA measurements with 0, 1, 2, and 3 heating cycles were immersed for 1 day in THF with a mass ratio of 1:20 of polymers to the solvent. The insoluble parts of the polymers were collected and the masses were compared to the mass of the original samples. The samples that were not analyzed by using DMA were totally soluble. The insoluble portion of the samples increased with the number of thermal cycles (Figure 18). These insoluble materials are cross-linked polymers which might be the products of the DA adduct aromatization or maleimide homopolymerization [48,58,59,60].

The procedures performed to study the occurrence of the DA chemistry in the polymers revealed information that are in agreement with one another. Changes in the relevant signals of the FTIR spectra indicated that the DA and rDA reactions occurred in the polymers, including the possible occurrence of the endo and exo stereoisomers. Moreover, the changes in the mechanical properties in the DMA curves were in agreement with the FTIR spectra. Both characterizations also suggested that both reactions took place repeatedly during repeated thermal cycles, nevertheless, the reversibility decreased during the measurement. The trend is also found in other studies involving the furan and maleimide pair, in which the DA adduct underwent aromatization or maleimides homopolymerization [48,52,58]. This conclusion is supported by the occurrence of insoluble solids in the samples after the DMA measurement, as both side reactions resulting in irreversibly cross-linked polymers [48,59,60]. The literature suggested that both aromatization and maleimide homopolimerization occur at elevated temperatures. Therefore, reprocessing the thermally reversible polymers at lower temperatures might reduce the formation of irreversible cross-links.

Further experiments were performed to explore the possibilities in applying the polymers into technical use. The polymers J-40-60 and SF-80-20 were used to bind two aluminum sheets. The bonding strength was examined according to ASTM D1876-08 with some modifications. The bonded sheets were pulled apart in a 180° direction using a tensile tester, and the average bonding strength was calculated. As a comparison, a commercial mounting glue was also tested using the same procedure.

**Figure 19:**
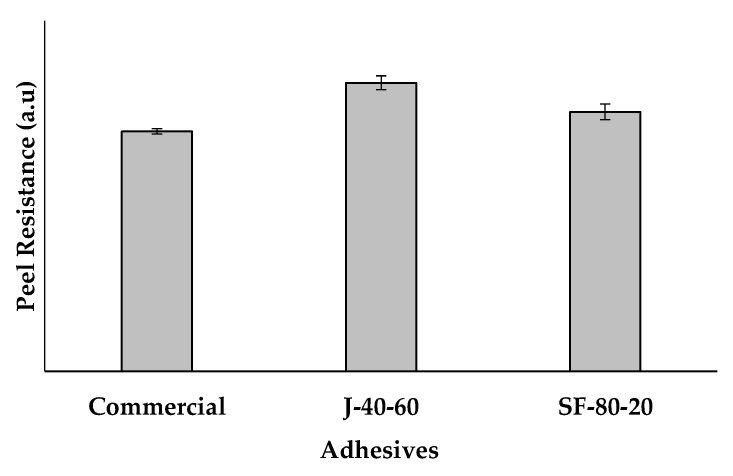
The average peel bonding strength of adhesives made of the polymers J-4-60 and SF-80-20, compared to a commercial mounting glue.

The average peel resistance of the adhesives is given in Figure 19. The peel resistance of J-40-60 and SF-80-20 exceeded the performance of the commercial mounting glue, therefore, the polymers can be considered as a potential adhesive material. Polymer J-60-40 showed higher bonding strength than SF-80-20. J-40-60 contained a higher concentration of the aromatic bismaleimide, thus it was more rigid than SF-8-20. This result is in agreement with a literature stating that the more flexible polymers usually perform lower cohesive strength than the rigid ones [62]. This result is an evidence of how the polymers are potentially applicable as adhesives, even without modification. The correlation between the structure and the bonding strength can be used as a guidance in designing the material to tune its performance.

## 4. Conclusions

Thermally reversible low cross-link density polymers were synthesized from vegetable oils containing different number of unsaturations, viz. jatropha and sunflower oils (3.6 and 4.5 C–C double bonds per triglyceride molecule). The oils were epoxidized to fully convert the double bonds into epoxide groups. The epoxidized oils were reacted with furfurylamine in order to attach furan groups into the structure according to an optimized condition developed for epoxidized jatropha oil. The condition ensured minimum conversion of the ester groups and maximum number of furans attached to the oil. However, the same condition did not work as well for epoxidized sunflower oil due to steric hindrance. There is more triglyceride structure retained in the jatropha-based monomer rather than in its sunflower counterpart (in average 2.36 compared to 2.00 esters/molecule), while the number of furans attached were less in the modified jatropha oil (2.57 compared to 3.42 furans/molecule).

Mixtures of aromatic and aliphatic bismaleimides were used to tailor the mechanical properties of the polymers, resulting in a series of polymers with different brittleness with T_g_ ranging from 3.6 to 19.8 °C. The properties were influenced by the structure of the furan-functionalized oil and the composition of bismaleimides. Sunflower-based polymers were more brittle with higher T_g_, due to their higher cross-link density. Higher aliphatic bismaleimide concentration lowered the T_g_ and induced higher flexibility of the polymers.

Changes in the chemical structure and mechanical properties observed in FTIR and DMA measurements suggest that the Diels-Alder and retro-Diels-Alder reactions occurred in recurring thermal cycles. However, an increasing part of the polymers were irreversibly cross-linked with more thermal cycles, which might be caused by aromatization of the DA adduct and homopolymerization of the maleimide groups.

The polymers J-40-60 and SF-80-20 were successfully applied as adhesives, as shown by the results of T-Peel Test. The bonding strength of these polymers were better than a commercial mounting glue, with J-40-60 showing the best performance. The rigidity of the polymers was found to correlate with the bonding strength of the adhesives.

All in all, the present study clearly demonstrates the possibility to cross-link vegetable oil derivatives in a thermally reversible way, as already demonstrated in many other works. The obtained polymers were found to be applicable as adhesives with better performances than a commercial mounting glue. However, the presented data also clearly indicate the occurrence of side reactions that are typical of this DA chemistry and adduct. These side reactions influenced the thermal and mechanical behavior. Future studies might address a quantitative evaluation of these side reactions and possibly their use for designing novel materials displaying the possibility to thermally reversible or irreversible cross-linking as a function of the curing temperature.

## Figures and Tables

**Table 1:** Composition of bismaleimides in different polymers.

Sample Codes	Vegetable Oil	Aliphatic Bismaleimide	Aromatic Bismaleimide
J-40-60	Jatropha	40%	60%
J-60-40	Jatropha	60%	40%
J-80-20	Jatropha	80%	20%
SF-60-40	Sunflower	60%	40%
SF-80-20	Sunflower	80%	20%
SF-100-0	Sunflower	100%	0

## Supplementary Materials

The following are available online at https://www.mdpi.com/2073-4360/12/8/1708/s1, Figure S1: FTIR spectra of the aromatic and aliphatic model compound, at 50 °C of the first heating; Figure S2: Storage modulus of the polymers, measured without material pockets; Figure S3: The storage modulus of polymer J-80-20 on the first heating and cooling cycle.
