# Supplementary material for: Thermally Reversible Polymeric Networks from Vegetable Oils
Source: Polymers (Basel). 2020 Jul 30;12(8):1708. doi: 10.3390/polym12081708 (PMC7465172; doi:10.3390/polym12081708)
Supplement: Supplementary file 1 [file polymers-12-01708-s001.pdf]

Supplementary Information : Thermally Reversible Thermosetsfrom Vegetable Oils

Frita Yuliati, Ranjita K. Bose, Jennifer Hong, Keshia S. Indriadi, and Francesco Picchioni

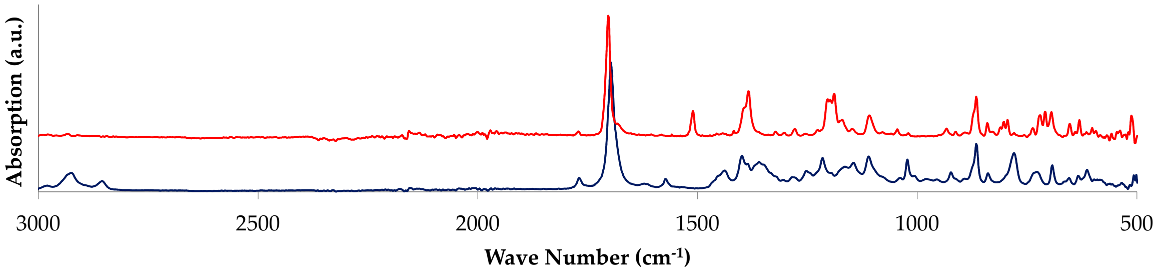

Figure S1. FTIR spectra of the aromatic and aliphatic model compound, at 50 °C of the first heating

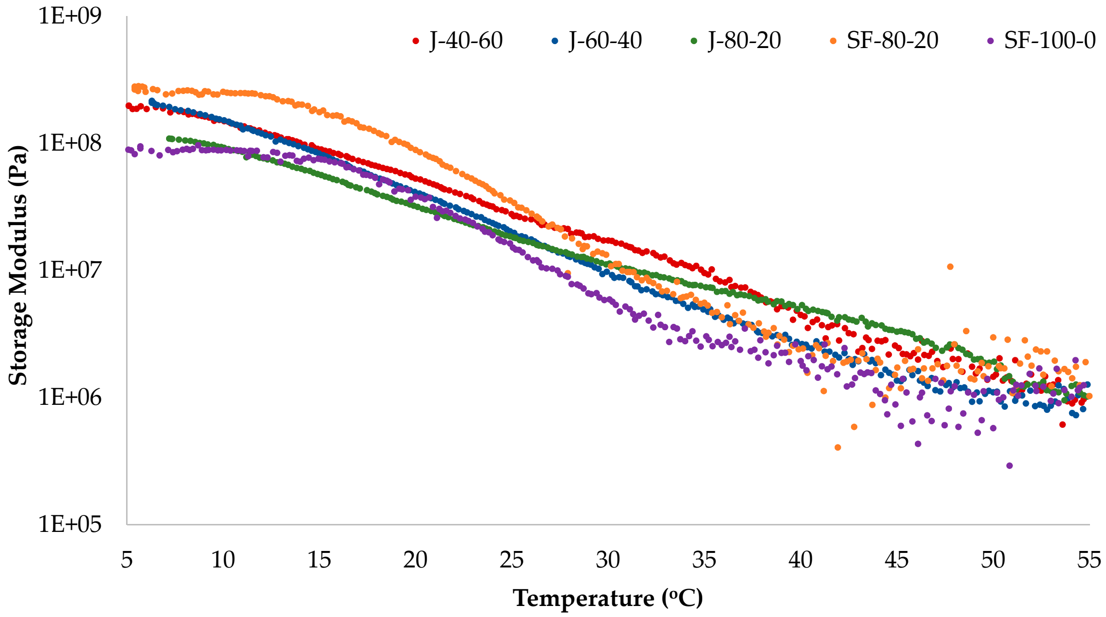

Figure S2. Storage modulus of the polymers, measured without material pockets.

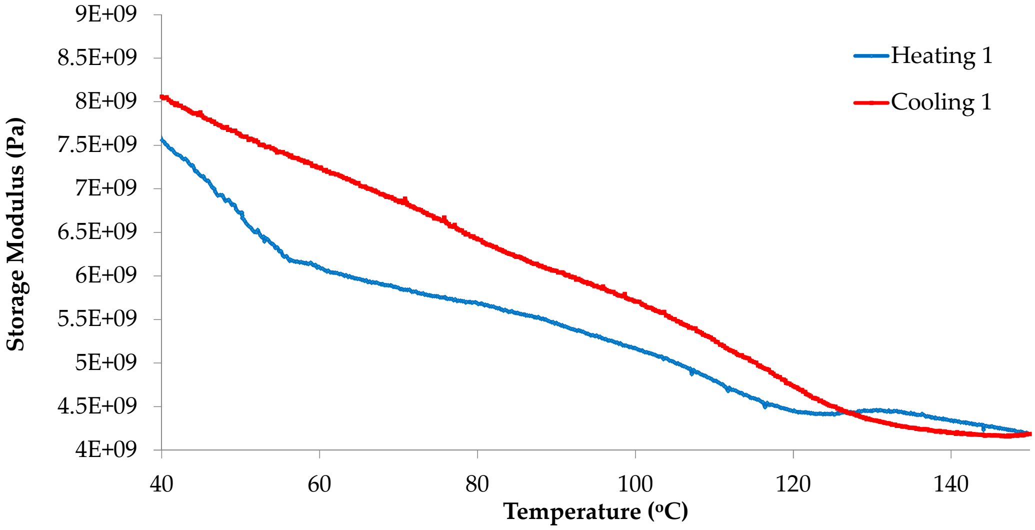

Figure S3. The storage modulus of polymer SF-100-0 on the first heating and cooling cycle. The measurement was performed by using a material pocket, thus the absolute value of the modulus includes that of the material pocket.
